# Guideline Compliance in Chronic Heart Failure Patients with Multiple Comorbid Diseases: Evaluation of an Individualised Multidisciplinary Model of Care

**DOI:** 10.1371/journal.pone.0093129

**Published:** 2014-04-08

**Authors:** Tam H. Ho, Gillian E. Caughey, Sepehr Shakib

**Affiliations:** 1 Department of Clinical Pharmacology, Royal Adelaide Hospital, Adelaide, South Australia, Australia; 2 Quality Use of Medicines and Pharmacy Research Centre, Sansom Institute, School of Pharmacy and Medical Sciences, University of South Australia, Adelaide, South Australia, Australia; Indiana University, United States of America

## Abstract

**Objective:**

To assess the impact of individualised, reconciled evidence-based recommendations (IRERs) and multidisciplinary care in patients with chronic heart failure (CHF) on clinical guideline compliance for CHF and common comorbid conditions.

**Design and setting:**

A retrospective hospital clinical audit conducted between 1^st^ July 2006 and February 2011.

**Participants:**

A total of 255 patients with a diagnosis of CHF who attended the Multidisciplinary Ambulatory Consulting Services (MACS) clinics, at the Royal Adelaide Hospital, were included.

**Main outcome measures:**

Compliance with Australian clinical guideline recommendations for CHF, atrial fibrillation, diabetes mellitus and ischaemic heart disease.

**Results:**

Study participants had a median of eight medical conditions (IQR 6–10) and were on an average of 10 (±4) unique medications. Compliance with clinical guideline recommendations for pharmacological therapy for CHF, comorbid atrial fibrillation, diabetes or ischaemic heart disease was high, ranging from 86% for lipid lowering therapy to 98% anti-platelet agents. For all conditions, compliance with lifestyle recommendations was lower than pharmacological therapy, ranging from no podiatry reviews for CHF patients with comorbid diabetes to 75% for heart failure education. Concordance with many guideline recommendations was significantly associated if the patient had IRERs determined, a greater number of recommendations, more clinic visits or if patients participated in a heart failure program.

**Conclusions:**

Despite the high number of comorbid conditions and resulting complexity of the management, high compliance to clinical guideline recommendations was associated with IRER determination in older patients with CHF. Importantly these recommendations need to be communicated to the patient’s general practitioner, regularly monitored and adjusted at clinic visits.

## Introduction

Chronic heart failure (CHF) occurs in 1.5–2.0% of Australians.[1] Its incidence and prevalence rise markedly with age; 10% in people aged ≥65 years to over 50% in people aged ≥85 years.[2]–[3] The presence of comorbidity is common in CHF patients, with a median of 6 comorbid conditions,[4] and those with high comorbidity accounts for the majority of inpatient hospital stays for CHF patients.[5] Highly prevalent cardiac comorbid conditions in patients with CHF include atrial fibrillation or flutter (AF), ischaemic heart disease (IHD), and diabetes mellitus (DM), which are present in 27–75% of patients with CHF.[6]–[8] The ageing population and associated increasing prevalence of comorbidity pose increasing complexity and challenges in applying clinical guidelines into practice. Most clinical guidelines are disease specific and often fail to address the needs of patients with multiple chronic conditions.[9]–[10] The use of disease specific guidelines for those with multiple chronic conditions may in fact be associated with detrimental effects, including difficult, complicated, inappropriate and harmful treatment regimens.[4], [9].

Multidisciplinary care has been recommended as best-practice management for patients with CHF.[1], [11] There is high level evidence that demonstrates for those hospitalised for HF, application of multidisciplinary programs of care significantly reduces all cause mortality, hospital re-admission, in addition to improving quality of life for patients and reduced health expenditure.[1], [12] Current evidence supports a number of key components of multidisciplinary care that can be grouped under four broad domains, including biomedical care, self-education and support, psychological care and palliative care, within which coordination of care and inclusion of agreed treatment and care goals are central throughout.[12] For older patients with multiple conditions, there is a clear need for a multi-disciplinary model of care which allows incorporation of patient preferences, individualisation of disease specific guideline recommendations, and reconciles differences and conflicts between them.[13] In this study we examined the effect of such a model of care, on clinical guideline compliance in patients with CHF, within the Australian setting. The influence of common comorbid conditions, including atrial fibrillation, ischemic heart disease and diabetes and other patient and clinic-related factors on clinician guideline compliance were also assessed.

## Methods

This study was approved by the Royal Adelaide Hospital Human Research Ethics Committee. Written consent was given by the patients for their information to be stored in the hospital database and used for research purposes.

### Study Sample

The study inclusion criteria were all patients with a documented clinical diagnosis of CHF who attended The Multidisciplinary Ambulatory Consulting Service (MACS) clinic at a tertiary teaching hospital from mid 2006 to February 2011. There were no exclusion criteria. For the purpose of this study, systolic heart failure (HF) was defined as HF with ejection fraction (EF) ≤40% according to the Heart Foundation Guidelines[1], in the absence of a quantitative assessment, a subjective report of moderate to severe left ventricular dysfunction. Patients without echocardiography, were assumed to have had systolic heart failure.

### Study Sample

The MACS is a holistic management model for patients with multiple comorbidities, that is based on multidisciplinary assessments and the determination of individualised, reconciled evidence-based recommendations (IRERs). All patients in the MACS clinic have a holistic assessment consisting of a self-administered questionnaire which covers living circumstances, activities of daily living, fall history, vaccination status, appetite and depression questionnaires. On a clinic visit, patients firstly have a nursing assessment consisting of an averaged sitting blood pressure (BP), a standing BP, and social assessment. They then undergo a medical review by a pharmacist before seeing a physician. The physician can then generate IRERs using a web-based database that includes the documented patient specific information, including comorbid conditions and uses an alogrithm to resolve variations between evidence-based recommendations between comorbid conditions and highlight conflict where resolution is not possible. This results in a reconciled list of evidence-based recommendations individualised specifically for each patient (Appendix S1). The recommendations are based on evidence-based management of CHF from Australian clinical guidelines including Heart Foundation guidelines[1], and Therapeutic Guidelines[14], [15], and also included evidence-based management of common comorbid conditions in CHF including atrial fibrillation, ischaemic heart disease and diabetes. The core evidence-based recommendations are seen as the minimum standard of care which should be considered for all patients attending the service with that condition. These recommendations are divided into different categories including pharmacological (ACE inhibitor and beta blocker therapy, blood thinning, blood pressure, lipid, and glycaemic control), lifestyle education (exercise, fluid intake, salt intake, performing daily weighs), investigations, referrals, action plans and vaccination. The recommendations based on the evidence based guidelines were then discussed and agreed upon by the patients and physicians. Responsibility for attaining these recommendations were determined by the clinician, some assigned to a member of the multi-disciplinary team or the patient’s general practitioners or the clinician themself.

### Determination of Clinician Guideline Compliance

Clinician guideline compliance criteria were developed *a priori* and for each patient their applicability, compliance or reason for non-compliance was assessed cross-sectionally across all patients in the clinic database as of August 2011, regardless of whether the patients had ongoing management throughout the clinic. Evidence based guideline recommendations examined for CHF included; use of CHF medicines, CHF medicine titrations, development of individual exercise program, heart failure education, influenza or pneumococcal vaccination, smoking intervention; anticoagulation for atrial fibriallation; HbA1c to target in diabetes, use of anti-platelets, lipids to target in diabetes, blood pressure to target in diabetes, eye check in diabetes; lipids to target, blood pressure to target and use of lipid lowering therapy in ischaemic heart disease. The association of compliance with these guideline recommendations and patients’ demographic data, patient-related factors (number of medicines, number of medical problems, Simplified Nutritional Appetite Questionnaire total[16], Geriatric Depression Score[17], and number of falls), and clinic-related factors (number of appointments, whether IRERs were documented or not, number of IRERs, primary physician, enrolment in a heart failure program, management by a cardiologist) extracted from the clinic database, were examined.

### Statistical Analysis

Data was analysed using SPSS, version 17.0. Continuous variables are presented as means ± standard deviations or as medians and corresponding 25^th^ and 75^th^ percentiles (interquartiles - IQR). Categorical variables are presented as absolute values and proportions of patients. To evaluate associations between comorbid conditions, patient demographics or system factors and guideline compliance we used student’s t test to analyse normally distributed data. We used the Mann-Whitney test for ordinal data. χ^2^- test was used to analyse nominal data. Probability values of p<0.05 were considered statistically significant.

## Results

### Baseline Patient Characteristics

A total of 255 patients with a diagnosis of CHF were eligible for inclusion in the study. Table 1 shows baseline patient characteristics. The median age was 81 years (IQR 75–86), approximately a third lived alone (37%). They had a median of 8 comorbid conditions (IQR 6–10) and were receiving on average 10 (SD±4) different medicines. They were often seen in multiple visits, 4 (IQR 2–8), cardiologists were primary physicians for 21% of patients while general physicians, geriatricians and clinical pharmacologists were for the remaining. At the time of the clinician guideline assessment, 207 patients (81.2%) had completed their management through MACS, and the remainder had ongoing appointments.

**Table 1:** Baseline Characteristics of CHF Patients (n = 255).

Characteristics	N (%) (unless stated otherwise)
*Demographics*	
Age (median, IQR)	81 (75–86)
Female	136 (53%)
Lives alone	94 (37%)
*Clinical characteristics*	
Systolic heart failure	109 (43%)
Number of documented medical conditions (median and IQR)	8 (6–10)
Patients with documented IRERs	235 (92%)
Number of medicines on presentation (mean and SD)	10±4
Total number of appointments	4 (2–8)
SNAQ total	14 (12–16)
Geriatric depression score	5 (2–8)
Seen by a cardiologist	63 (21%)
Seen by a consultant	246 (96%)
Falls (total patients)	196
0 falls	106 (54%)
1–2 falls	63 (32%)
>2 falls	27 (14%)

IQR, Interquartile range; IRERs, individualised reconciled evidence-based recommendations; SNAQ, Simplified Nutritional Appetite Questionnaire.

### Clinician Guideline Compliance

Table 2 shows compliance with clinical guidelines for each management criteria for CHF, atrial fibrillation, diabetes and ischaemic heart disease. This included those patients that did not have any contraindications for guideline recommended management strategies. In general there was very good compliance with medication management for CHF and associated comorbid therapies, with values ranging from 86% for lipid therapy in those with comorbid ischaemic heart disease to 97% for RAS antagonists.

**Table 2:** Compliance with clinical guideline recommendations for CHF patients.

*Management criterion*	*Patients should have the management N* †	*Compliance N (%)*
**Chronic heart failure n = 255**		
RAS antagonist	90	87 (97%)
Maximized dose		82 (94%)
Betablockers	98	91 (93%)
Maximized dose		87 (96%)
Exercise program	195	70 (36%)
Heart failure education	226	170 (75%)
Influenza vaccination	208	170 (82%)
Pneumococcal vaccination	183	116 (63%)
**Atrial fibrillation n = 122**		
Anticoagulation	79	69 (87%)
**Diabetes mellitus n = 114**		
HbA_1_c to target	114	92 (81%)
Any lipid therapy	68	62 (91%)
Lipids to target		50 (74%)
BP to target	100	91 (91%)
Ophthalmology review	69	24 (35%)
Podiatry review	109	0 (0%)
**Ischaemic heart disease n = 147**		
Anti-platelet	139	136 (98%)
Any lipid therapy	94	81 (86%)
Lipids to target		69 (73%)
BP to target	130	122 (94%)
Smoking intervention	10	9 (90%)

† *number represents total number of patients with indication and no contraindication for guideline based management.*

**Figure 1:**
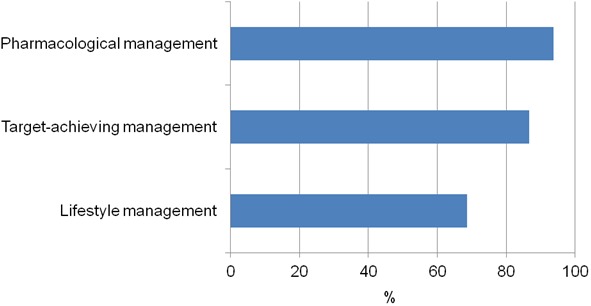
Proportion of CHF patients compliant with clinical guidelines recommendations grouped lifestyle, target-achieving and pharmacological management.

Individualised exercise program, HF education, vaccinations, ophthalmology review, podiatry review, achieving lipids to targets were the least compliant criteria. When these guideline management criteria were grouped according to lifestyle, target-achieving or medication management, compliance was highest (93.7%) with medication management (Figure 1).

### Patient and Clinic-related Factors Affecting Clinician Compliance with Guideline Based Recommendations

Associations between the patient demographics and clinical characteristics and clinician compliance with guideline management criteria for HF and comorbid conditions are shown in Table 3. IRER documentation was significantly associated with clinician compliance with all guideline recommendations studied, except for anticoagulation therapy in AF or the use of lipid lowering therapy in those with comorbid ischaemic heart disease (Table 3). Similarly increasing numbers of IRERs set was associated with increased clinician compliance with having an individualised exercise program, HF education, influenza and pneumococcal vaccination, anticoagulation for comorbid AF and HbA1c and lipids to target levels in comorbid diabetes. Enrolment in a HF program was associated with higher compliance with HF specific recommendations, including individualised exercise programs, HF education and influenza vaccination. Higher numbers of healthcare appointments was also associated with better clinician compliance with having an exercise program, HF education, vaccinations and meeting HbA1c to target levels in comorbid diabetes (Table 3). All other patient and clinical characteristics investigated were not significantly associated with any of the guideline management criteria studied (data not shown).

**Table 3:** Factors significantly affecting compliance with clinical guideline recommendations.

Patient or Clinical Characteristic	Guideline Recommendation		p value
	**Exercise (n = 195)**		
	Compliant (n = 70)	Non-compliant (n = 125)	
Age (Median, IQR)	79 (71–84)	82 (75–86)	0.036
Enrolment in heart failure program	33 (47%)	25 (20%)	<0.001
IRERs determined	68 (97%)	107 (86%)	0.012
Number of IRERs (Mean ± SD)	24±10	17±11	<0.001
Number of appointments (Median, IQR)	6 (4–11)	3 (2–7)	<0.001
	**Heart failure education (n = 226)**		
	Compliant (n = 170)	Non-compliant (n = 56)	
Enrolment in heart failure program	73 (43%)	1 (2%)	<0.001
IRERs determined	167 (98%)	39 (70%)	<0.001
Number of IRERs (Mean ± SD)	24±20	10±9	<0.001
Numbers of appointments (Median, IQR)	5 (3–10)	2 (1–3)	<0.001
Management by a cardiologist	48 (28%)	4 (7%)	0.001
Lives alone	54 (32%)	28 (50%)	0.017
	**Influenza vaccination (n = 208)**		
	Compliant (n = 170)	Non-compliant (n = 38)	
Age (Median, IQR)	80 (78–86)	79 (69–84)	0.02
Enrolment in heart failure program	59 (35%)	4 (11%)	0.003
IRERs determined	161 (95%)	27 (71%)	<0.001
Number of IRERs (Mean ± SD)	22±10	11±11	<0.001
Numbers of appointments (Median, IQR)	5 (3–10)	2 (1–3)	<0.001
	**Pneumococcal vaccination (n = 183)**		
	Compliant (n = 116)	Non-compliant (n = 67)	
IRERs determined	108 (93%)	55 (82%)	0.027
Number of IRERs (Mean ± SD)	24±10	17±11	<0.001
Numbers of appointments (Median, IQR)	5 (3–11)	3 (2–5)	<0.001
	**Anticoagulation for AF (n = 79)**		
	Compliant (n = 69)	Non-compliant (n = 10)	
Age (Median, IQR)	81 (76–85)	85 (81–94)	0.025
Number of IRERs (Mean ± SD)	20±11	9±9	0.004
	**Meet HbA1c target in diabetes (n = 114)**		
	Compliant (n = 92)	Non-compliant (n = 22)	
IRERs determined	90 (98%)	15 (68%)	<0.001
Number of IRERs (Mean ± SD)	25±9	17±13	0.011
Numbers of appointments (Median, IQR)	5.5 (3–11)	3 (1–6.3)	0.021
	**Lipids to target in diabetes (n = 68)**		
	Compliant (n = 50)	Non-compliant (n = 18)	
IRERs determined	46 (92%)	13 (72%)	0.048
Number of IRERs (Mean ± SD)	25±11	15±12	0.007
Numbers of appointments (Median, IQR)	6 (2.8–11)	3 (1.8–4.3)	0.028
	**Blood pressure to target in diabetes (n = 100)**		
	Compliant (n = 91)	Non-compliant (n = 9)	
IRERs determined	85 (93%)	6 (67%)	0.033
	**Eye checked in diabetes (n = 69)**		
	Compliant (n = 24)	Non-compliant (n = 45)	
IRERs determined	24 (100%)	36 (80%)	0.022
Management by a cardiologist	46%	20%	0.03
	**Lipid lowering therapy in ischemic heart disease (n = 94)**		
	Compliant (n = 81)	Non-compliant (n = 13)	
Age (Median, IQR)	79 (74.5–83)	83 (79–88.5)	0.014

IRERs, individualised reconciled evidence-based recommendations.

## Discussion

The results of this study demonstrate that a multidisciplinary model of care, utilising individualised, reconciled evidence-based recommendations, is associated with high clinician compliance to clinical guideline recommendations, for older patients with HF, despite the high number of comorbid conditions and resulting complexity of management and care. The focus on a list of holistic evidence-based recommendations which are individualised for the patient, within the context of all medical conditions present, provides the opportunity to focus on the individual needs of the patient, rather than individual disease states and for the inclusion of patient preferences when making treatment decisions. Whilst current Australian guidelines recommend multidisciplinary care for people with HF[12], there has been no study to date formally examining the effects of such care on concordance with guidelines recommendations, particularly for those with multiple comorbid conditions. This is the first study to examine clinical guideline compliance for CHF and comorbid conditions, in a multidisciplinary model of care.

The clinician compliance with guideline recommendations using this model of care for these complex patients, was better than that reported in previous studies focused on managing isolated CHF. In the IMPORVE HF study[18] in outpatient cardiology practices in US, compliance with RAS antagonists was 79%, beta-blockers was 87.6%, HF education was 60.7%, and with anticoagulation in AF was 70%. In a European study[6] across 24 countries of 3658 patients with a diagnosis of left ventricular systolic dysfunction, they found compliance to RAS inhibitors and beta blockers was between 80–86% and 42–63%, respectively. In the CASE study[19] on 2905 CHF patients in Australian general practices, rates of ACEI, ARB, and beta-blocker uses were 70.7%, 6.4% and 13.9%, respectively. In a study of CHF inpatients admitted to the same venue as the current study, at the time of discharge, 59% of patients were on ACEIs, and 43% were on beta-blockers. These patients were not seen in cardiology or MACS clinics.[7] ^.^ Although multidisciplinary care and an increased awareness of the benefits of CHF management may have contributed to our results, a consistent finding in our data was the positive association between the determination of IRERs and clinician guideline compliance. The determination of the IRERs, and their modification and further individualisation based on patient preferences, has many benefits in the management of multimorbid patients. This includes a basis for discussion of patient centred goals and patient preferences; provision of decision support for the large number of diverse evidence-based recommendations for complex patients; it acts as a prompt and a checklist for keeping patients on track for achieving treatment outcomes when they have disease exacerbations or admissions which may interrupt their routine outpatient management. In addition, the high adherence to guideline recommendations (both pharmacological and non-pharmacological) in our study and a willingness to participate in healthier behaviours may have been facilitated by the multidisciplinary model of care, together with the provision of IRERs. Medication adherence has previously been reported as a marker for adherence to other treatments or behaviours that may affect health outcomes.[20], [21].

In our current study, clinician adherence to pharmacological treatment recommendations was greater than that observed for lifestyle measures. Often lifestyle consultations consume greater time and effort than starting patients on medicines and commencement of pharmacological therapies may also be better documented than lifestyle measures. The MACS clinic was developed by the Clinical Pharmacology department, in the study hospital, as an identified need to manage polypharmacy and the intervention gap in HF management, hence medications are a focus. The outcomes may have been different if a dietician or exercise physiologist was a member of the team instead of a pharmacist. Further, recommendations requiring the achievement of specific targets which generally require multiple steps such as achieving BP, lipid or glycated Hb targets, were not performed as well as single-step interventions. Another factor which appeared to influence guideline compliance ambiguity of whether the primary responsibility for an intervention sat within the MACS clinic or in primary care, for example vaccinations and referrals to ophthalmologists and podiatrists. None of diabetic patients had a documented podiatry review however, this was not included in our clinic core recommendations. This highlights the role of the recommendations in acting as a reminder for guideline based management, as well as helping support documentation of outcomes.

Whilst we observed that IRER determination within multidisciplinary care was associated with increased compliance with guideline recommendations, the number of visits was also significantly associated with improved clinician uptake of guideline recommendations. When visits reached five to six times, many additional core managements occurred. This highlights the complexity of this patient population, and the fact that in order to achieve evidence-based recommendations, a substantial clinical investment has to be made.

The study highlighted areas where care that could be improved. Dedicating a nurse to initiate lifestyle measures may bring better adherence into these aspects. Although increased number of clinic visits was associated with higher guideline compliance in many aspects, patients who are compliant are likely to be seen more times and more likely to have all of their recommendations met. Managing patients with multiple comorbidities as it is largely a step-wise process. Each change usually occur one step at a time. A system for documentation of the change and communication to other team members is vital especially as the process of care is often interrupted by disease exacerbations, and other social and psychological interferences.

Our study has several limitations. The study was based on a clinic database, making it difficult to compare with previous studies where mainly case-note reviews were used. Due to the design of the MACS clinic and the current study, whereby there were no exclusion criteria for the cohort selection, it was not possible to compare our results with an internal control group. The clinic attracts referrals of complex multimorbid patients with CHF and a comparable cohort of patients in other hospital outpatient clinics who were not referred to the MACS clinic were not able to be identified. Furthermore, the cohort of patients in the MACS clinics do not represent a general outpatient population. These patients selectively have multiple comorbid conditions, where multidisciplinary team and multiple appointments are probably more useful in this setting, and this may have biased the referral patterns for patients more likely to respond. The provision of care received within the hospital setting is likely to be different to that received within community settings and as a result patients may have been more likely to participate in self-care, resulting in improved outcomes.

In conclusion, provision of multi-disciplinary care utilising individualised reconciled evidence-based recommendations for older patients with CHF and multiple comorbid conditions, resulted in high clinician compliance with clinical guideline recommendations. In an era of increasing focus on patient-centred care, the inclusion of patient preference and circumstance in formulating goals for healthcare is of increasing importance, particularly for those with multiple conditions.

## Supporting Information

Appendix S1 Determination of individualised reconciled evidence-based recommendations.
(DOC)
