# Supplementary material for: Guideline Compliance in Chronic Heart Failure Patients with Multiple Comorbid Diseases: Evaluation of an Individualised Multidisciplinary Model of Care
Source: PLoS One. 2014 Apr 8;9(4):e93129. doi: 10.1371/journal.pone.0093129 (PMC3979669; doi:10.1371/journal.pone.0093129)
Supplement: Appendix S1 — Determination of individualised reconciled evidence-based recommendations. (DOC) [file pone.0093129.s001.doc]

| Condition | Recommendation category | Intensity | Recommendation | Target |
| --- | --- | --- | --- | --- |
| Atrial fibrillation | Blood thinning | 40 | Patient should be offered anticoagulation unless contraindicated |  |
| Chronic heart failure | ACE inhibitor | 30 | Patients with systolic heart failure should be considered for maximum dose ACE inhibitor (or AII blocker if appropriate) unless contraindicated |  |
| Chronic heart failure | Beta blocker |  | Patients with systolic heart failure should be considered for maximum tolerated dose of beta blocker unless contraindicated |  |
| Chronic heart failure | Exercise | 50 | Refer patient to formal exercise program |  |
| Chronic heart failure | Influenza vaccination |  | Patient should have yearly influenza vaccination |  |
| Diabetes | Blood pressure | 40 | Control BP to target depending on presence of proteinuria | <130/80 or <125/75 if proteinuria exceeding 1gm/day |
| Diabetes | Blood thinning | 10 | Patients should be considered for antiplatelet therapy |  |
| Diabetes | Exercise | 20 | Encourage the patient to undertake regular physical exercise |  |
| Diabetes | Influenza vaccination |  | Patient should have yearly influenza vaccination |  |

***Appendix S1. Determination of individualised reconciled evidence-based recommendations***

Example using comorbidities of atrial fibrillation, chronic heart failure, and diabetes, and categories of blood thinning, ACE inhibitor and beta-blocker therapy, exercise, blood pressure management, and influenza vaccination.

For each of the commonly occurring medical condition seen in the clinic, a set of core evidence-based recommendations are developed based on national guidelines.^1, 14, 15^  The core evidence-based recommendations are seen as the minimum standard of care which should be considered for all patients attending the service with that condition. These recommendations are divided into different categories including pharmacological (ACE inhibitor and beta blocker therapy, blood thinning, blood pressure, lipid, and glycemic control), lifestyle education (exercise, fluid intake, salt intake, performing daily weighs), investigations, referrals, action plans, vaccination.

For each condition and category a recommendation is determined, as in the example above. If different conditions had different recommendations for the same category e.g. diabetes and chronic heart failure for exercise category above, the recommendations are compared, and an ordinal scale of intensity was developed with a higher number indicating a higher intensity. In the example above, a recommendation for heart failure patients of referral to a formal exercise program with an exercise physiologist, has a higher intensity, than encouragement to undertake regular exercise for patients with diabetes. Similarly conditions with a lower blood pressure target were given a higher intensity than those with a higher target.

Patients with systolic heart failure should be considered for maximum tolerated dose of beta blocker unless contraindicated.

For other recommendation categories such as influenza vaccination different conditions resulted in exactly the same recommendation i.e. yearly vaccination, hence there was no need to develop an intensity ranking for these categories.

The clinician is able to automatically generate these individualized reconciled evidence-based recommendations using the web-based clinic database. The database then looks to the patient’s documented clinical conditions and relevant recommendations, run an algorithm to reconcile the recommendations, and display the reconciled list individualized for the patient’s comorbidities.

The algorithm was thus:

- where only a single recommendation is made for a recommendation category, then that recommendation is displayed in the list
- where multiple recommendations are made for the same recommendation category without an intensity score e.g. influenza vaccination, then this recommendation would be displayed to the clinician only once in the recommendations list
- where multiple recommendations are made for the same recommendation category with an intensity score, the database displays the recommendation and target for the most intensive recommendation i.e. with the highest intensity score

Hence in the example above the following recommendations would be made for a patient with atrial fibrillation, chronic heart failure and diabetes:

- Patient should be offered anticoagulation unless contraindicated
- Patients with systolic heart failure should be considered for maximum dose ACE inhibitor (or AII blocker if appropriate) unless contraindicated
- Patients with systolic heart failure should be considered for maximum tolerated dose of beta blocker unless contraindicated
- Refer patient to formal exercise program
- Patient should have yearly influenza vaccination
- Control BP to target depending on presence of proteinuria

Although this system is able to reconcile similar recommendations for different goals, it does not take into account the fact that some conditions may be a contraindication to treatment recommendations for another condition e.g. postural hypotension and intensive blood pressure lowering.

Hence the database algorithm also included a list of condition-recommendation pairs, where a contraindication existed, with an explanatory text. The recommendations list is developed in the same way as described above, however, if a contraindicating condition existed for the recommendation, then the whole recommendation appears in red, and a roll-over text explains the reason for caution.

The following shows what how the above recommendations would appear for a patient who also has a history of falls, with the explanatory text appearing as a roll-over when the mouse hovers over the question mark for recommendation 2 regarding anticoagulation.


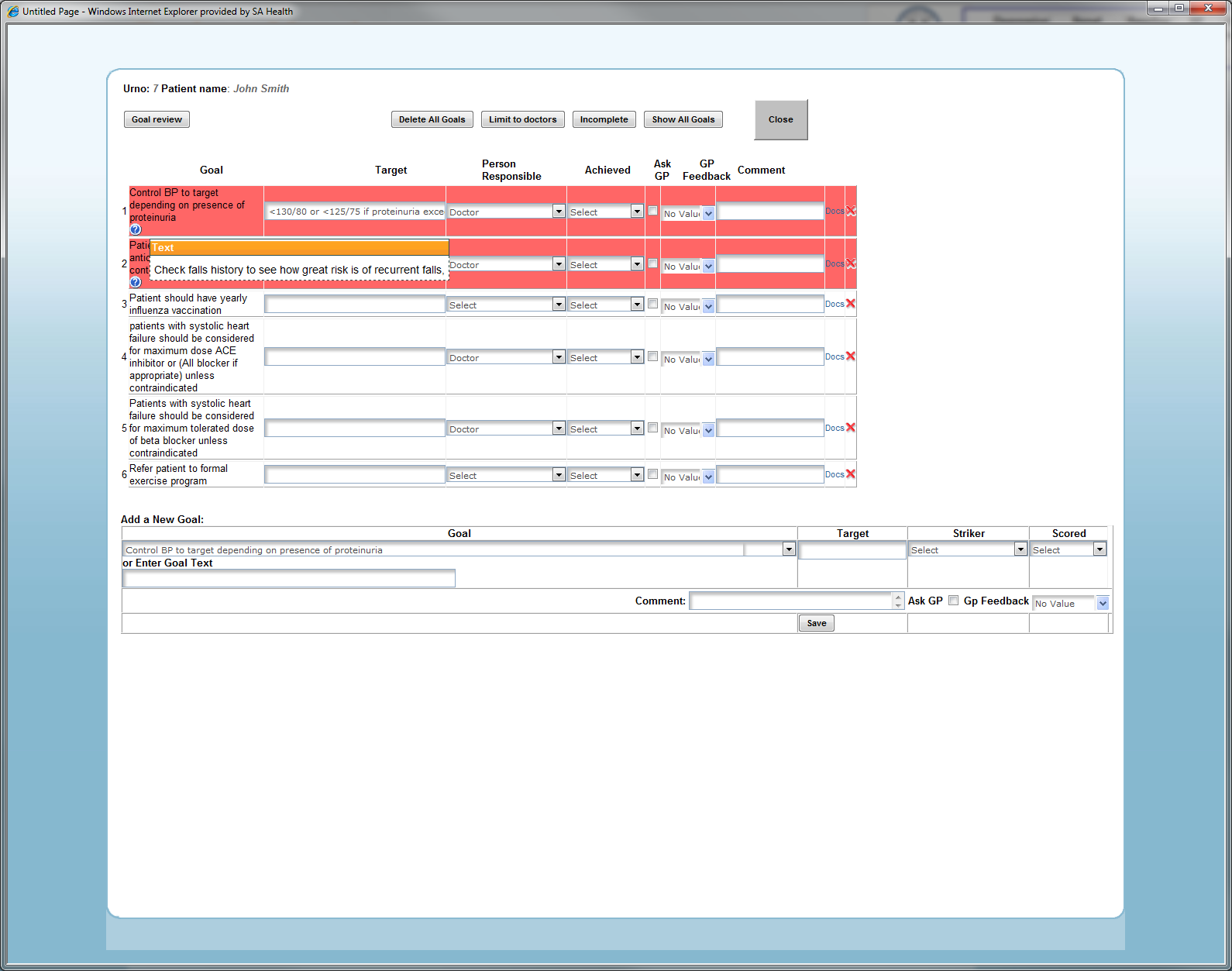


Individual recommendations can be added in as free text for the patient as well, and any of the automatically generated goals can be modified to individualise them for the patient.
